# Clinical Significance of Körner’s Septum in Relation to Occurrence of Squamous Chronic Otitis Media

**DOI:** 10.7759/cureus.31070

**Published:** 2022-11-03

**Authors:** Mithula Murali, Shraddha Jain, Vaidehi Hande

**Affiliations:** 1 Otolaryngology - Head and Neck Surgery, Jawaharlal Nehru Medical College, Datta Meghe Institute of Medical Sciences (Deemed to be University), Wardha, IND

**Keywords:** cholesteatoma, otosurgery, temporal bone, petrosquamosal lamina, körner’s septum, chronic otitis media

## Abstract

The Körner's septum (KS), persistent petrosquamosal lamina, is a bony lamina (developmental remnant) that extends from the articular fossa to the mastoid apex, above the middle ear, and runs inferiorly and laterally to the facial nerve canal. The petrous and squamous bones meet at this septum. The anatomical structure of KS, which most frequently occurs at the level of the head of the malleus and/or the anterior semicircular canal, is described in depth in this work. The embryological elements of the temporal bone development that result in the formation of KS are taken into consideration. Clinically KS is considered an important anatomical variation, in the development of chronic diseases of the ear like chronic otitis media, especially attic retraction pockets, and cholesteatoma as it can contribute to attic blockage. Also, studies have found a significant association between tympanosclerosis and KS. High-resolution computed tomography (HRCT) and cone-beam computed tomography are the two imaging methods most commonly used to identify KS. It is observed that KS was associated with an increased risk for chronic otitis media, and residual cholesteatoma. The purpose of this review article is to provide a general overview of the KS and its clinical implication, as well as to summarize and discuss the latest clinical data regarding this entity.

## Introduction and background

The Körner's septum (KS) is an osseous lamina that divides the mastoid's air cells within the temporal bone. In 1887, Hartmann described KS as a thin bony wall that lies in between the antrum petrosum and the antrum mastoideum. Later scholars noted its presence, but it was not until Körner that the clinical importance of the septum was brought up [1]. Advancements in temporal bone surgery sparked a rise in interest in KS. A prominent KS may be mistaken for the bony lamina covering the sigmoid sinus, according to Shulman and Rock [2]. The potential of mistaking the KS for the medial wall of the antrum is now highlighted by researchers [3-5].

The KS is regarded as a component of the petrosquamosal suture since it is situated at the point where the petrous and squamous portions of the temporal bone meet. The petrosquamosal suture is divided into three segments, with different names in different publications, according to the literature [6-8]. The most popular classification is that proposed by Virapongse et al. [8]. This allows for the distinction of the anterior (ventral), middle, and posterior (dorsal) sections. Also considered to be a component of the KS (middle/tympanic portion) is a cog, a bony ridge in the epitympanum [8].

The KS extends from the petrosquamosal fissure in the posterior part of the mandibular fossa, travels over the middle ear cavity (slightly slanted from the front and side, in the posterior-medial direction, within the tegmental wall), and then directs downward, laterally to the facial nerve canal (in its vertical - mastoid part), and into the direction of the mastoid apex. Mastoid cells are divided into deep and superficial cells by the posterior portion of KS. According to Proctor B, KS continuity may be broken at multiple points [6]. The septum is most frequently observed in relation to anatomical markers at the levels of the head of the malleus and the superior semicircular canal, whereas it is least frequently seen at the level of the tympanic sinus [9]. Unfortunately, it is still unclear whether the discontinuity of the septum is due to abnormal development or injury from disease-related damage. A study by Ozer et al. showed that the prevalence of KS was higher in patients with Tympanosclerosis and they recommended mastoidectomy to be performed in cases of severe tympanosclerosis in order to remove KS [4]. The presence of KS might also increase the risk of residual cholesteatoma [4]. An inexperienced otologist may damage the facial nerve in the presence of a complete KS [4]. The purpose of this review article is to provide a general overview of the KS and its clinical implication, as well as to summarize and discuss the latest clinical data regarding this entity.

## Review

Anatomy of KS 

Mastoid cells are divided into deep and superficial cells by KS, which corresponds to the persistent petrosquamosal suture [2]. Analysis of high-resolution computed tomography (HRCT) scans of the temporal bone reveals that it is equally prevalent on both sides, with a preference for men [10].

Wojciechowski et al. state that the KS is divided into three sections: the anterior, which is the most constant and is located at the level of the head of the malleus, the superior, which is located at the level of the superior semicircular canal, and the posterior (the least constant, at the level of the tympanic sinus) [7]. Some writers argue that the tympanic region of the septum (midportion) is an osseous ridge known as a cog because KS may vary in form and/or manifest with discontinuities/disruptions [8]. The cog separates the epitympanum into two parts: the anterior section, also known as the supratubal recess, and the posterior part, which is located within the superior wall of the tympanic cavity. It is interesting to note that all patients with cogs also had KS at the mastoid antrum [4]. Inflammatory processes emerge as a result of decreased ventilation of the tympanic cavity between the protympanum and antrum, which is caused by the middle part of the septum.

Squamous chronic otitis media and KS

According to the Browning classification, there are two forms of squamous COM: inactive (squamous) COM-pars tensa/flaccida retraction and retained debris. Additionally, there is an active (squamous) COM with pars flaccida/tensa retraction, squamous-type epithelial debris, and inflammatory, pus-filled middle ear mucosa [10]. KS can indirectly cause posterosuperior or attic retracting of the TM, by decreasing ventilation, and can result in otitis media [11]. It has been established that mastoid pneumatization and the degree of tympanic membrane retraction have a relationship, and cholesteatoma is observed to be more common in poorly pneumatized mastoids [12]. According to one study, the cholesteatoma can develop in well-pneumatized mastoids [13]. Additionally, whether primary or secondary sclerosis is to blame for cholesteatoma remains a mystery. The majority of COM cases have secondary sclerosis because the new bone is being formed [14]. KS, a developmental remnant between the temporal squama and the mastoid (petrosquamosal suture persistence), is found to be one of the factors affecting the etiopathogenesis of COM [14].

Diagnostic imaging of KS

**Figure 1:**
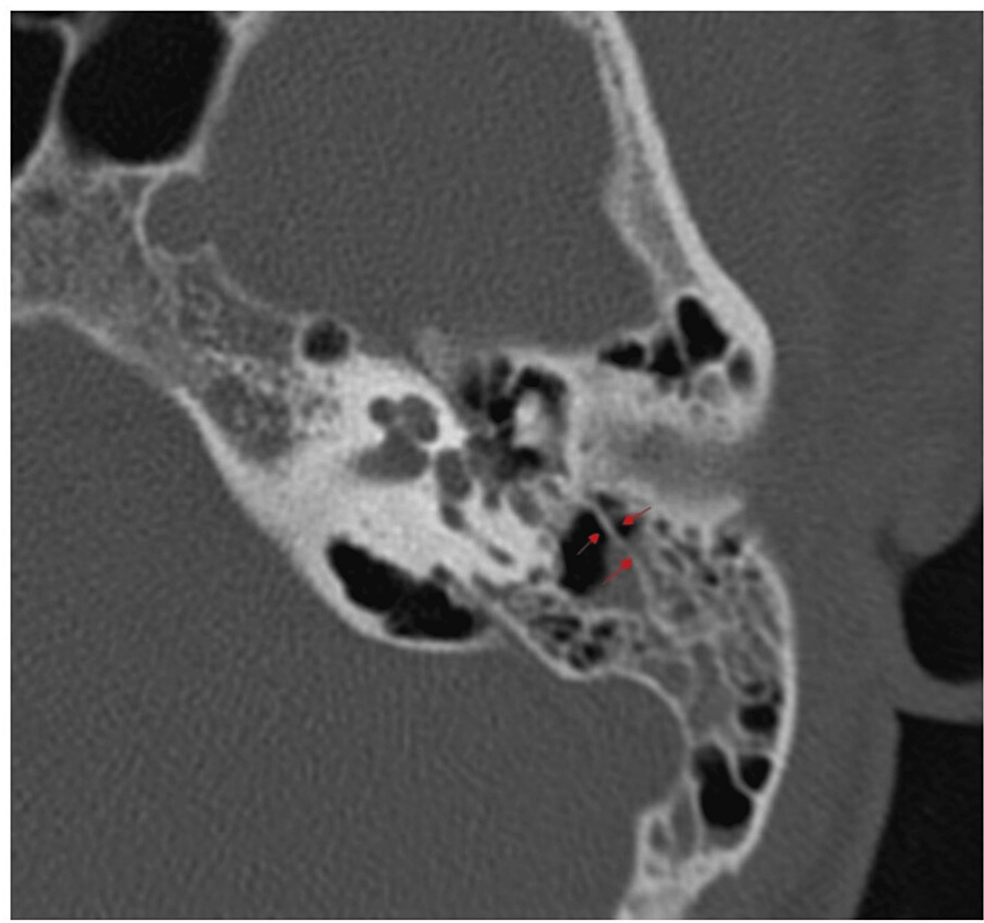
HRCT temporal bone: axial section with right ear showing the presence of Korner's septum (red arrows)

**Figure 2:**
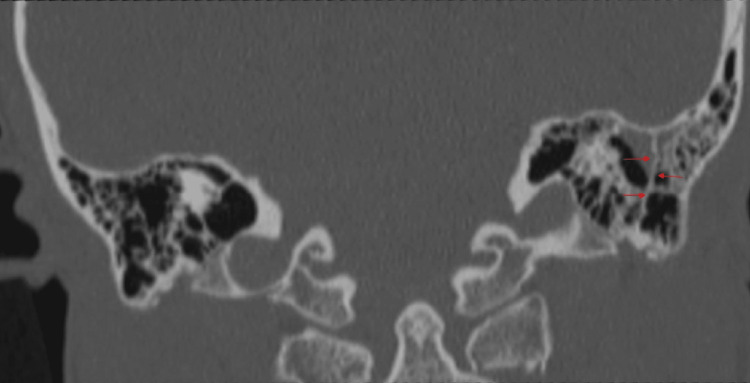
HRCT temporal bone: coronal section with right ear showing the presence of Korner's septum (red arrows)

HRCT and cone-beam computed tomography (CBCT) of the temporal bone provides the clearest images of KS [7,15]. With typical coronal reconstructions in HRCT, data collecting is often carried out in the axial plane, parallel to the lateral semicircular canal [15]. It is seen as a bony thickening in the axial section (Figure 1) and coronal section (Figure 2). The field of vision is limited to the area between the level of the superior mastoid cells and the level of the stylomastoid foramen, as well as the upper wall of the external auditory canal. After obtaining images of both temporal bones in a soft tissue window with a slice thickness of 1.0-1.25 mm to examine the soft tissues next to the bone and the posterior fossa, each temporal bone is independently reconstructed in a bone window with a slice thickness of no more than 1 mm (it usually ranges 0.4-0.6 mm) [15]. It is common practice to perform HRCT on the temporal bones without using a contrast agent. CBCT is evolving into a different imaging technique from traditional CT scanning [16]. It makes use of a revolving gantry with a connected x-ray tube and detector. Through the temporal bone's center, a cone-shaped x-ray beam impinges on a two-dimensional (2D) x-ray detector. To acquire a volumetric data set that enables additional multiplanar reconstructions with slice thickness in the range of 0.3-1 mm using specialized software, a single 360° gantry rotation is required [17].

Clinical relevance of KS

KS could be a serious clinical issue. The antrum and epitympano-mastoid canal appear to be a false medial wall during antromastoidectomy [4,5]. The pneumatized portion of the temporal bone contains a closed area that, if KS is not opened during antrum exploration, may result in inflammation and a return of the chronic otitis media.

It also has a big impact on middle ear surgery since there is a higher probability of leaving behind residual cholesteatoma in the cog-covered posterior portion of the epitympanum. Therefore, it is recommended to conduct a mastoidectomy with anterosuperior tympanostomy in cases of broad KS [4]. In patterns with substantial KS, co-existing structural anomalies such as infantile mastoid, sigmoid sinus near to the ear canal, like in the example we described, and hypoplastic antrum have also been recorded [18]. However, the HRCT study found no association between the size of KS and mastoid pneumatization [17].

The prevalence of KS in healthy ears was reported to be 6.58%, compared to 30.4% in ears with retraction pockets and/or tympanic membrane adhesion, and 17.4% in cases of chronic otitis media without retraction pockets by Göksu et al., who did not validate these findings [18,19]. In 28% of the 356 ears that underwent tympanoplasty, according to Karaca et al., and in 24% of the operated temporal bones, according to Toros et al. [15,20]. In 25%-33% of the temporal bones that were investigated, partial KS was present [7,18]. According to certain theories, the perimatrix of a growing cholesteatoma may react with proteolytic enzymes, which could partially destroy the KS while paradoxically increasing pneumatization of the ear and preventing the development of retraction pockets and tympanosclerosis. Tympanosclerosis with adhesive otitis media is more common than chronic otitis media with or without cholesteatoma. Tympanosclerosis with adhesive otitis media is characterized by obstruction of ventilation of the antrum and/or antrum and epitympanum induced by the presence of KS. Additionally, KS is documented in cases with tympanosclerosis more frequently than in cases of any other kind of chronic otitis media [4]. In light of these findings, KS may result in chronic inflammation by obstructing airflow between the protympanum and the antrum as well as between the antrum and the remaining mastoid cells [4].

Additionally, the bone plate known as the KS, which separates the mastoid cells, is called the petrosquamous lamina (at antrum). KS is more persistent in adults than in children in the temporal bone. In a few investigations, KS was observed in chronic otitis media with squamosal disease [21]. Middle ear ventilation being obstructed by the anatomical blockage is another reason that may contribute to the development of COM. Researchers Jain et al., Methwani et al., and Singh et al. have published studies on chronic otitis media and also found that KS can be one of the factors affecting the etiopathogenesis of chronic otitis media [22-24]. Few more related research was reviewed [25].

## Conclusions

This study provides a better understanding of one of the important etiopathogenesis associated with COM especially the squamosal type, the KS. The causes of the displacement of the squamous portion of the growing temporal bone that results in KS during the second and third trimesters of pregnancy are yet unknown. However, credible research findings and a sizable number of clinical case reports support the hypothesis that the presence of KS increases the risk of developing inflammatory middle ear conditions like cholesteatoma, retraction pocket, and tympanosclerosis, likely as a result of decreased ventilation of the middle ear cavities. Clinically, it is crucial to perform thorough pre-surgical imaging diagnostics of the temporal bones using HRCT and/or CBCT. These tests enable otorhinolaryngologists to determine whether KS is present and, if it is, to categorize it as a complete or partial septum. However, more investigations are necessary to determine the impact of KS, a developmental variant and a serious clinical issue, on the course of middle ear inflammatory processes naturally.
